# Glutamate-Mediated Primary Somatosensory Cortex Excitability Correlated with Circulating Copper and Ceruloplasmin

**DOI:** 10.4061/2011/292593

**Published:** 2011-11-21

**Authors:** Franca Tecchio, Giovanni Assenza, Filippo Zappasodi, Stefania Mariani, Carlo Salustri, Rosanna Squitti

**Affiliations:** ^1^Laboratory for Electrophysiology for Translational neuroScience (LET'S), Istituto di Scienze e Tecnologie della Cognizione (ISTC), Consiglio Nazionale delle Ricerche (CNR), Fatebenefratelli Hospital Isola Tiberina, Rome, Italy; ^2^Neurology, Department of Imaging, San Raffaele Cassino, Rome, Italy; ^3^Department of Clinical Neuroscience, Institue of Neurology, Campus Bio-Medico of Rome University, Rome, Italy; ^4^Department of Neuroscience and Imaging, G. D'Annunzio University, Chieti, Italy; ^5^Associazione Fatebenefratelli per la Ricerca biomedica (AFaR), Department of Neuroscience, Fatebenefratelli Hospital Isola Tiberina, Rome, Italy

## Abstract

*Objective*. To verify whether markers of metal homeostasis are related to a magnetoencephalographic index representative of glutamate-mediated excitability of the primary somatosensory cortex. The index is identified as the source strength of the earliest component (M20) of the somatosensory magnetic fields (SEFs) evoked by right median nerve stimulation at wrist. *Method*. Thirty healthy right-handed subjects (51 ± 22 years) were enrolled in the study. A source reconstruction algorithm was applied to assess the amount of synchronously activated neurons subtending the M20 and the following SEF component (M30), which is generated by two independent contributions of gabaergic and glutamatergic transmission. Serum copper, ceruloplasmin, iron, transferrin, transferrin saturation, and zinc levels were measured. *Results*. Total copper and ceruloplasmin negatively correlated with the M20 source strength. *Conclusion*. This pilot study suggests that higher level of body copper reserve, as marked by ceruloplasmin variations, parallels lower cortical glutamatergic responsiveness.

## 1. Introduction

In the last decade, growing evidence has unveiled the involvement of specific metals in brain cortical neurotransmission and the role played by their disarrangements in neurodegeneration. It is well established, for example, that cognitive impairments often follow conditions of metal imbalance, which can be either deficiency, as in Menkes' disease, or accumulation, as in Wilson's disease or aceruloplasminemia [1–6]. Recently, it has been demonstrated that metal dyshomeostases linked to mutations and polymorphisms of specific genes, such as ATP7B in Alzheimer's disease (AD, [7]), the gene of ceruloplasmin in Parkinsons' disease (PD, [8, 9]), the gene of hemochromatosis (HFE) in multiple sclerosis [10, 11] and in AD [12–14], increase the risk of developing those diseases. Copper and zinc have been advocated as primary actors in the neurotransmission of glutamatergic synapses in brain areas that are critical for AD [5, 15, 16]. Iron appears instead mostly involved in dopaminergic neurotransmission [17]. 

Most of the data on the role of metals in neurotransmission come from studies *in vitro* or animal models, while data in humans are still rather scanty. For this reason, in the current study, we approached the issue in man aiming at assessing whether systemic concentrations of copper, iron, and their related proteins are associated with specific brain indices of cortical glutamatergic neurotransmission. 

Glutamate mediates the excitatory neurotransmission in brain networks that are key for sensory perception, memory, and sensorimotor control. In particular, glutamate is the excitatory neurotransmitter of the thalamocortical inputs to the primary visual, auditory, and somatosensory cortices [18, 19], which makes it crucial for the cortical hierarchic structure controlling the interactions with the environment. 

We focused on the primary somatosensory cortex (S1, [20]), since it receives the incoming stimulus via a simple circuit (relayed by only two subcortical nuclei in the brain stem and thalamus) and used a median nerve stimulation protocol. It is known that the earliest cortical response to peripheral nerve stimulation, the M20, results from the glutamate-mediated excitatory postsynaptic potentials (EPSPs, [21–23] generated by area 3b's pyramidal neurons [24–26]. The fact that M20 is highly reliable and is not affected by the subject attention [27] makes its ECD strength a good index of glutamate-mediated excitability in clinical studies, where patient compliance could be limited.

We used MEG to record cerebral activity. Selective sensitivity to electrical currents directed tangentially to the scalp [28] and the cytoarchitectonic distribution of pyramidal cells columns (perpendicular to the cortical surface) make MEG highly sensitive to the activity of area 3b [29–31]. We used localization algorithms to assess the amount of synchronously activated neurons independently of their position. In other words, we wanted the estimate of glutamate-mediated excitability independent of both the recording apparatus positioning with respect to the subject and the individual central sulcus shaping with respect to the scalp. 

The aim of our work was twofold: (1) to assess noninvasively in healthy people possible relationships between glutamate-mediated neuronal excitability and metal known to affect this neurotransmission path. (2) To explicate the existence of a noninvasive index of S1 glutamate-mediated excitability (M20 ECD strength), whose reliability will be indirectly strengthened by the ability to assess such relationships.

## 2. Materials and Methods

The study was conformed with The Code of Ethics expressed in the Declaration of Helsinki and was approved by the Ethical Committee of the “San Giovanni Calibita” Fatebenefratelli Hospital. All subjects signed an informed consent. 

### 2.1. Subjects

Thirty healthy and drug-free subjects (16 females, mean age 51 ± 22, age range [24–93] years) were enrolled in the study. All were right handed with a mean Edinburgh Inventory test score of 83 ± 4 [32]. Neurological history and examination were assessed to exclude sensory deficit. Electroneurographic study of upper extremities was conducted to exclude subclinical deficit of sensitive fibres. Twenty-seven out of the 30 recruited had a complete data set that entered the statistical analysis.

### 2.2. Biochemical Investigations

Samples of serum from overnight fasting blood were drawn in the morning and rapidly stored at −80°C. Biochemical variables were determined according to established methods reported in details elsewhere [33]. Briefly, serum copper concentrations were measured following the method of Abe et al. [34] (Randox Laboratories, Crumlin, UK) and by an A Aanalyst 300 Perkin Elmer atomic absorption spectrophotometer equipped with a graphite furnace with platform HGA 800. Transferrin [35] and ceruloplasmin [36] were measured by immunoturbidimetric assays (Horiba ABX, Montpellier, France). 

**Figure 1:**
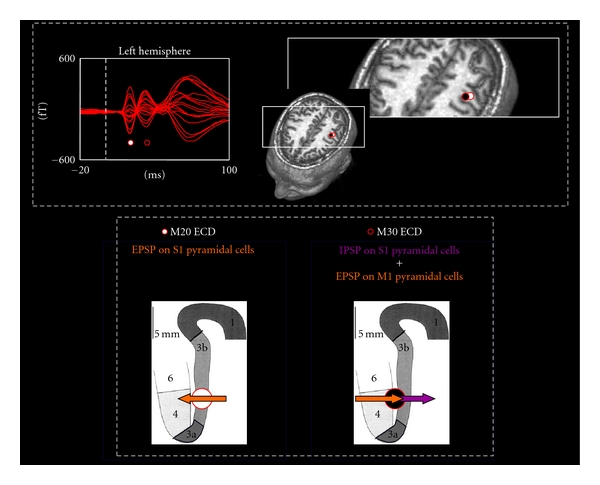
MEG primary sensorimotor cortex excitability indexes. Top left: in a representative subject, the superimposition of all left rolandic region channels in the [−20, 100] ms period, 0 being the moment of the galvanic stimulus arrival to the right median nerve at wrist. Top right: the position of the ECDs explaining the cerebral activation in correspondence to the M20 (white circle) and M30 (black circle) components is projected onto a suitable axial magnetic resonance image slice, through integration on the basis of individual anatomical landmarks. Bottom: schematic representation of currents subtending M20 and M30 generators in a suitable sagittal section of primary sensory and motor areas. Orange arrow indicates current induced by EPSP and purple arrow the effect of IPSP. Circles represent the position of the corresponding equivalent current dipole. The M20 component is mainly generated by EPSPs impinging on pyramidal neurons in area 3b and is mediated by glutamate neurotransmitters. The currents associated with the IPSPs impinging on pyramidal neurons in 3b area are mediated by GABA neurotransmitter and are added to the EPSPs onto BA4 pyramidal neurons to give rise to M30 component.

Serum iron levels were determined using a Ferene colorimetric method (Horiba ABX, Montpellier, France) [37]. TF saturation (%TF-sat) was calculated by dividing serum iron by the total iron-binding capacity (TBC = TF in mg/dL ∗ 1.25) and multiplying by 100. Zinc level was measured using the specific complexant 5-Br-PAPS [(2-5-bromo-2-pyridylazo)-5-(N-propyl-N-sulfo-propylamino) phenol] according to manufacturer instructions (Zinc, Sentinel Diagnostic, Milan, Italy) (Figure 1). 

All biochemical measures were automated on a Cobas Mira Plus analyser (Horiba ABX, Montpellier, France) and performed in duplicate. For each serum copper (total copper) and ceruloplasmin pair, we computed the amount of copper bound to ceruloplasmin (Cu_B_) and the amount of copper not bound to ceruloplasmin (free copper) following standard procedures [38]; briefly: Cu_B_ = ceruloplasmin (mg/dL)  ∗  10  ∗  *n*; *n* = 0.0472 (*μ*mol/mg); free copper = total  copper − Cu_B_). This calculation expresses free copper in *μ*mol/L and is based on the fact that ceruloplasmin contains 0.3% of copper [38]. Thus, for a subject with a serum copper concentration of 17.3 *μ*mol/L and a serum ceruloplasmin concentration of 33 mg/dL, the bound copper concentration = 33 ∗ 10 ∗ 0.0472 = 15.6 *μ*mol/L, and the free copper concentration = 17.3−15.6 = 1.7 *μ*mol/L.

### 2.3. MEG Investigation

Brain magnetic fields were recorded from the left rolandic region by means of a 28-channel MEG system [39] covering a scalp area of about 180 cm^2^, operating inside a magnetically shielded room (Vacuumschmelze). Cortical evoked responses were recorded (band-pass filtered 0.48–250 Hz, sampling rate 1000 Hz) during unilateral 0.2 ms long electric pulses (631 ms interstimulus interval) of the right median nerve at wrist, delivered through surface disks with proximal cathode. Stimulus intensity was adjusted until inducing a painless thumb twitch. In this way, all the proprioceptive and the superficial perception fibres are engaged, and neural recruitment is mainly dependent on measurable functional/anatomical circuitry. Moreover, the standardization of stimulus intensity just above motor threshold has been demonstrated to be able to evidence interindividual relationships of primary somatosensory cortical response amplitude with age and gender [40]. The electrical stimulation was delivered to the dominant right median nerve and the cerebral activity recoded contralaterally. 

The MEG signals from each of the 28 channels were averaged on the galvanic nerve stimulation at wrist (*t* = 0, about 300 responses) obtaining the SEFs. The primary cortical responsiveness was estimated by latency, position, and strength of the sources activated in correspondence to the two earliest components (M20 and M30), modelled by equivalent current dipoles (ECDs) within a homogenously conducting sphere, and solution accepted only if explained variance was above 95%.

Our main target was the ECD strength, representing the number of synchronously activated neurons [28], estimated by the localization algorithm in parallel to the ECD position, so that the strength estimate is independent of the source position with respect to the recording apparatus.

### 2.4. Statistical Analysis

All MEG and biochemical variable did not differ from a Gaussian distributions (Kolmogorov-Smirnov test, *P* > 0.200 consistently). 

To make the analysis independent of personal data, correlation with age was checked by computing Pearson coefficients. Gender dependence of M20 and M30 ECD strengths was evaluated by a multivariate analysis of variance with *component* (M20 and M30) as within-subject factors and *gender* (women and man) as between-subject factor. Independent-sample *t*-test was used to estimate the dependence on gender of the biochemical variables. The association of metal metabolism factors and primary somatosensory neural excitability was studied by correlation matrices between biochemical variables and left M20 and M30 ECD strengths after correction for dependence on age or gender when necessary. Bonferroni correction for multiple comparisons was applied.

## 3. Results

We controlled MEG indices and biometal variables for gender effects, but none was found (between-subject factor *gender P* > 0.200). Thus, descriptive values are provided as a whole (Table 1). M20 ECD strength (*r* = 0.403, *P* = 0.027), iron (*r* = −0.478, *P* = 0.009), and transferrin (*r* = −0.431, *P* = 0.014) were instead correlated for age. Thus, age dependence of M20 ECD strengths, iron, and transferrin levels was considered in all statistical analyses, and partial correlations were computed to correct for the age effect. Positions of either M20 ECD or M30 ECD (Table 2) displayed no dependence on age or gender. No correlations were found between the M20 and M30 strengths (*P* > 0.200). 

The assessment of the biological variables under study indicated that the healthy subjects evaluated in this panel had values coherent with normal reference ranges reported in literature (Table 1). Our statistical analyses revealed that among the biological variables under study, only copper correlated with M20 ECD strength (Table 3). In particular, higher levels of copper corresponded to lower M20 strengths. 

**Figure 2:**
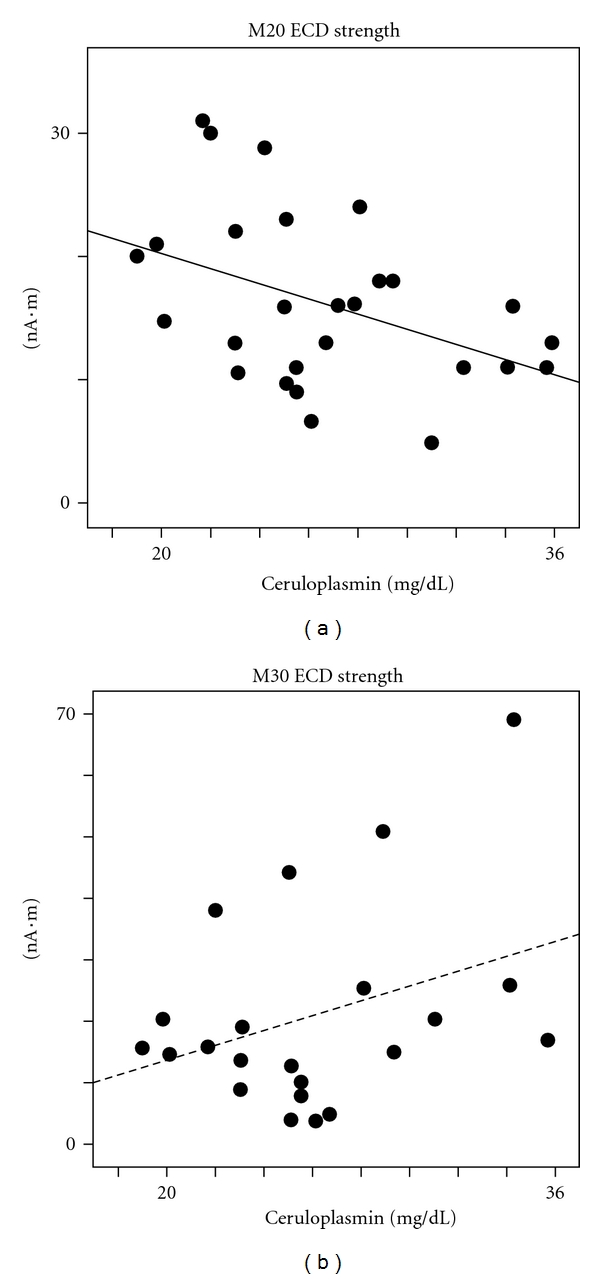
Ceruloplasmin and S1 cortex excitability. Scatter plot of M20 and M30 ECD strengths with respect to individual ceruloplasmin circulating levels. Regression lines are shown for significant (solid line) and nonsignificant (dashed line) correlations.

We also examined the relation between MEG indices and the two main components of serum copper: ceruloplasmin and free copper (see Section 2). M20 ECD strength did not correlate with free copper, but it displayed a strong inverse correlation with ceruloplasmin (Figure 2), similarly to serum copper (Table 3). Transferrin levels only showed a trend to inverse correlation with the sole M20 ECD strength, even though they did not approach significance (Table 3).

## 4. Discussion

The pilot investigation presented in this paper aimed at evaluating the involvement of systemic biometal-related variables in human neurotransmission. We focused on a specific brain circuit which connects pyramidal neurons in the somatosensory cortex with a projection coming from neurons in thalamus. Our main result is that in healthy subjects, the strength of M20's ECD, which we deem to be a good marker of glutamate-mediated cortical excitability, is associated with the serum concentrations of ceruloplasmin, which is a marker of copper status. 

### 4.1. Higher Copper Levels Associated with Lower Glutamatergic Excitability

The influence of copper on cortical glutamatergic transmission has been extensively demonstrated at a synaptic level, as copper has been shown to act as a high-affinity NMDA receptor blocker in a voltage-dependent manner [41]. Copper—through the Menkes adenosine triphosphatase (ATPase)—is directly involved in both *α*-amino-3-hydroxy-5-methyl-4-isoxazolepropionic acid receptor (AMPA) and N-methyl-D-aspartic acid (NMDA) glutamatergic receptors modulation, resulting in the inhibition of glutamate-mediated neurotransmission [15, 16, 42, 43]. In diverse methodological settings (rat olfactory bulb, [44] long-term potentiation (LTP) in rat hippocampus [45], copper has been demonstrated to be released from copper-containing vesicles in the synaptic cleft postsynaptically, after NMDA receptor activation, causing a depression or a complete blockage of the glutamate-mediated neurotransmission. ATPase7A, also known as the Menkes protein, was shown to be crucial for glutamatergic neurotransmission modulation. This copper chaperone was deemed to pump and accumulate copper into synaptic vesicles, replenishing a pool of copper to be released upon NMDA receptor stimulation. Copper released in the synaptic cleft functionally blocks NMDA receptor limiting Ca^2+^ entry and depolarization of the postsynaptic element [41, 45–47]. Additional copper proteins, such as mettallothiens-3, CuZn superoxide dismutase, and cytochrome, have been reported to modulate glutamatergic synaptic activity and to sustain neurotransmission. 

Finally, a similar mechanism was reported in other types of synapses, as, for example, those regulated by the P2X family receptors, which form nonselective channels activated by extracellular ATP. These receptors are widely expressed in the CNS and are involved in synaptic plasticity and in long-term potentiation. In particular, P2X4 receptor activity is inhibited by Cu^2+^ [48]. On the presynaptic side, prior protein (PrPc, [49]) is involved in regulating the presynaptic Cu^2+^ concentration and synaptic transmission linked to P2X receptors [50].

### 4.2. Ceruloplasmin and Copper Body Reserve

In normal conditions, about 85–95% of serum copper is structurally bound to ceruloplasmin, whereas the remainder is loosely bound to and is exchanged among albumin, *α*2 macroglobulin, amino acids, peptides, and several micronutrients and for this reason, is generally referred to as “free” copper [38, 51]. It is generally assumed that ceruloplasmin and free copper are more informative about, respectively, copper bioavailability or copper toxicity than the levels of serum copper. Ceruloplasmin is considered a reliable marker of body copper status since an increase in hepatic copper results in a sustained increase in serum ceruloplasmin concentrations [51–53]. Conversely, copper deficiency results in a ceruloplasmin serum decrease [53]. However, this notion does not hold during infancy, when the liver generates mostly apoceruloplasmin, which is the form of that protein which fails to incorporate copper during its synthesis in the liver, and it is rapidly catabolized [53]. On this ground, ceruloplasmin levels are largely used to monitor the effects of decoppering treatments, as, for example, in zinc therapy, or of chelating agents, as in Wilson's disease, representing a noninvasive surrogate marker of the body copper reserve [6, 54]. Free copper has, instead, been advocated as a superior diagnostic tool to detect copper toxicity, as in Wilson's disease, which is the quintessential example of copper toxicosis or accumulation [38].

### 4.3. Copper versus Iron: Relationship with Glutamatergic Excitability

If on the one hand copper status variations seem to account for the association of ceruloplasmin-MEG indices, on the other hand iron variations could be also seen as an explanatory reason of this association. In fact, iron metabolism cannot be isolated from copper homeostasis, since there is a crosstalk between copper and iron, represented specifically by ceruloplasmin. Ceruloplasmin is a key enzyme in iron metabolism, which oxidizes Fe^2+^ to Fe^3+^, thus, facilitating the transfer of cell iron to circulating [55]. In this framework, of particular interest is the cytochrome oxidase [56], which is an iron depending enzyme for energy production in the respiratory electron transport chain of mitochondria, which has been advocated as an endogenous metabolic marker of neuron typology, tightly coupling neuronal activity to oxidative energy metabolism [57]. However, the fact that iron, transferrin (corresponding to the total iron-binding capacity), and transferrin saturation (corresponding to the total bound iron), which are all markers normally used for diagnosis of diseases related to iron metabolism, show no correlation with M20's ECD strength speaks in favor of a primary role of copper in the glutamatergic neurotransmission-ceruloplasmin association.

### 4.4. M20 ECD Strength as Marker of S1 Glutamate-Mediated Excitability

Certainly, defining M20's ECD strength as a marker of glutamate-mediated neurotransmission implies a simplification. However, while previous evidence sustaining this notion was based on historical cyto- and circuit-architecture [24], more recent data have also been based on pharmacological manipulations of neuronal activities [58]. In particular, the administration of the GABA_A_ agonist lorazepam failed to attenuate the M20 strength [58]. Conversely, the strength of M30, which partially relies on GABA neurotransmission as demonstrated by its marked reduction upon lorazepam administration [58], did not associate with markers of copper metabolism in our study. Further investigations by proper pharmacological modulation of glutamate-mediate excitability should be performed to strengthen the concept of M20 ECD strength as noninvasive marker of this neurotransmission system. It is worth noticing that copper density was demonstrated in animal models to be higher in S1 than in all other cortical areas [59], suggesting a possible special role of copper in this primary cortical district. Whether the involvement of copper in glutamate-mediated neurotransmission found in S1 can be generalized to other cerebral regions requires devoted investigations.

To better understand the cause-effect nature of the relationship between glutamate excitability and copper, we plan to act on two levels: (1) in healthy subjects, collecting M20 ECD strength values before and after pharmacological modulation of specific neurotransmission systems (gabaergic transmission by diazepam/lorazepam/zolpidem; less clear how to modulate selectively glutamatrgic transmission, since topiramate, which inhibits glutamate transmission, seems to act also enhancing gabaergic transmission at least at the level of the human motor cortex) and (2) in patients who suffer from an altered copper metabolism, verifying the modulation of the copper-glutamate relationship.

### 4.5. Glutamatergic Excitability and Copper Status in Man

Human studies of copper homeostasis and of changes in the function of glutamate transporters have been performed in neurological diseases, whereas there is a lack of data from healthy subjects. To the best of our knowledge, a single study used transcranial magnetic stimulation (TMS) to explore cortical excitability and proton magnetic resonance spectroscopy (MRS) to asses metal-related metabolic function in amyotrophic lateral sclerosis [60, 61].

## Figures and Tables

**Table 1:** Metals/metal-related proteins.

	Study panel	Reference values
Copper [*μ*mol/L]	13.61 (2.53)	11–24.4
Ceruloplasmin [mg/dL]	26.29 (4.90)	20–60
Free copper [*μ*mol/L]	1.20 (1.96)	<1.6
Zinc [*μ*g/dL]	85.32 (14.75)	68–107
Iron [*μ*g/dL]	98.68 (32.92)	37–164
Transferrin [g/L ]	2.76 (0.41)	2–3.6
Transferrin saturation [%]	28.2 (8.4)	12–50%

Mean (standard deviation) of metals and metal-related proteins in serum.

**Table 2:** M20 and M30 ECD positions and strengths.

	*x* [mm]	*y* [mm]	*z* [mm]	*s* [nA · m]
M20	−42	−13	69	17.6
ECD	(10)	(11)	(13)	(9.3)
M30	−38	−12	71	21.5
ECD	(9)	(9)	(10)	(16.3)

Mean (standard deviation) position (*x*, *y*, *and* *z*) and strength (*s*) of left M20 and M30 ECDs. Coordinate system is defined on the basis of anatomical landmarks so that central axis passes through the midline between the two hemispheres, the positive *y*-axis passes through the nasion and the midpoint between the two preauricular points, and the positive *z*-axis passes through the vertex perpendicular to *y*-axes; thus, the positive *x*-axis is rightward.

**Table 3:** Correlation between SM1 excitability and metals/metal-related proteins.

	M20 ECD strength	M30 ECD strength
Copper	−**0.643** (<.0001)	0.185 (.435)
Ceruloplasmin	−**0.559** (.003)	0.303 (.194)
Free copper	−0.195 (.340)	−0.131 (.560)
Zinc	0.060 (0.808)	−0.015 (0.952)
Iron	−0.134 (0.522)	−0.002 (0.994)
Transferrin	−0.379 (0.068)	−0.065 (0.786)
Transferrin saturation	−0.23 (0.918)	0.080 (0.746)

Correlations (and statistical significance *P*) between primary sensorimotor cortex excitability and metals or metal-related proteins in serum. Note that correlation values are equal for ceruloplasmin-bound copper and ceruloplasmin (see Section 2). In bold correlation values with *P* < .005 (Bonferroni correction for multiple comparisons).
